# Supplementary material for: Comparative transcriptome analysis to identify the important mRNA and lncRNA associated with salinity tolerance in alfalfa
Source: PeerJ. 2024 Oct 16;12:e18236. doi: 10.7717/peerj.18236 (PMC11490228; doi:10.7717/peerj.18236)
Supplement: Supplemental Information 1 [file peerj-12-18236-s001.docx]

**The statistical power of this experimental design, G3__150_ vs G3__CK_, G5__150_ vs G3__150_, G5__150_ vs G5__CK_, G5__CK_ vs G3__CK_ calculated in lncRNASeqPower is 0.0566, 0.0593, 0.0633, 0.0600 and mRNASeqPower is 0.1649, 0.1881, 0.1640, 0.1454, respectively.**

**Table A1-1** Calculation of the power analysis of lncRNA-seq

| Comparison | Sequence depth | Dispersions | Sample number | Effect | false_positive_rate | Sequence power |
| --- | --- | --- | --- | --- | --- | --- |
| G3__150_vsG3__CK_ | 253.02 | 1.84 | 2,2 | 2 | 0.05 | 0.0566 |
| G5__150_vsG3__150_ | 255.09 | 1.73 | 2,2 | 2 | 0.05 | 0.0593 |
| G5__150_vsG5__CK_ | 215.39 | 1.60 | 2,2 | 2 | 0.05 | 0.0633 |
| G5__CK_vsG3__CK_ | 213.33 | 1.71 | 2,2 | 2 | 0.05 | 0.0600 |

**Table A1-2** Calculation of the power analysis of mRNA-seq

| Comparison | Sequence depth | Dispersions | Sample number | Effect | false_positive_rate | Sequence power |
| --- | --- | --- | --- | --- | --- | --- |
| G3__150_vsG3__CK_ | 166.09 | 0.70 | 2,2 | 2 | 0.05 | 0.1649 |
| G5__150_vsG3__150_ | 166.90 | 0.64 | 2,2 | 2 | 0.05 | 0.1881 |
| G5__150_vsG5__CK_ | 171.68 | 0.70 | 2,2 | 2 | 0.05 | 0.1640 |
| G5__CK_vsG3__CK_ | 170.87 | 0.76 | 2,2 | 2 | 0.05 | 0.1454 |
